# Supplementary material for: Repetitive concussions promote microglia-mediated engulfment of presynaptic excitatory input associated with cognitive dysfunction
Source: Commun Biol. 2025 Feb 28;8:335. doi: 10.1038/s42003-025-07729-1 (PMC11871131; doi:10.1038/s42003-025-07729-1)
Supplement: Supplementary file 1 — Supplementary Information [file 42003_2025_7729_MOESM1_ESM.docx]

**Supplementary Information**

**Repetitive concussions promote microglia-mediated engulfment of presynaptic excitatory input associated with cognitive dysfunction.**

Maryam Chahin^1,2,3^, Julius Mutschler^1,2,#^, Stephanie P Dzhuleva^1,2,#^, Clara Dieterle^1,2^, Leidy Reyes Jimenez^1,2^, Srijan Raj Bhattarai ^1,2^, Valerie Van Steenbergen^1,2^ and Florence M Bareyre*^1,2,4^

1 Institute of Clinical Neuroimmunology, University Hospital, LMU Munich, 81377 Munich, Germany

2 Biomedical Center Munich (BMC), Faculty of Medicine, LMU Munich, 82152 Planegg-Martinsried, Germany

3 Graduate School of Systemic Neurosciences, LMU Munich, 82152 Planegg-Martinsried, Germany

4 Munich Cluster of Systems Neurology (SyNergy), 81377 Munich, Germany

# Contributed equally

* Corresponding author. Email: florence.bareyre@med.uni-muenchen.de

ORCID Florence M Bareyre: 0000-0002-0917-1725


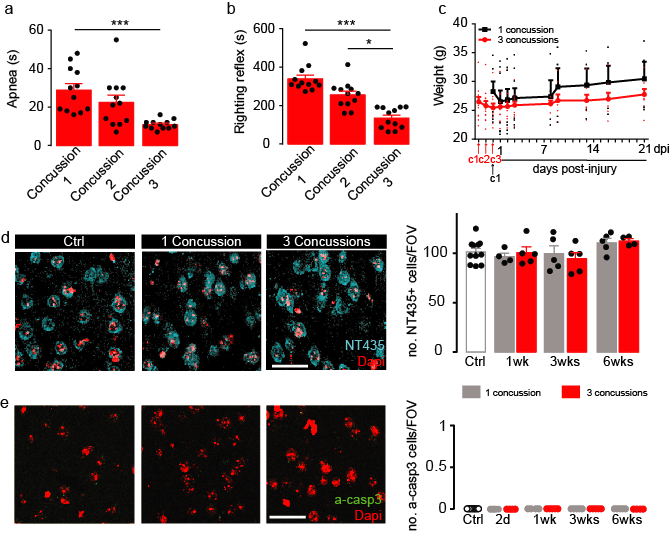


**Supplementary Figure 1. Repetitive concussions do not trigger cell loss or cell death.** (**a**) Quantification of apnea duration following repetitive concussions. p=0.0003 one-way ANOVA and Dunnett’s test 1st vs 3rd concussion. (**b**) Quantification of righting reflex following repetitive concussions. p<0.0001 1st vs 3rd contusion and p=0.0479 2nd vs 3rd concussion using a Kruskal-Wallis and Dunn’s test. (**c**) Quantification of the animal weight following single or repetitive concussions over a 21d period. Arrows indicate the time of concussions (c1/c2/c3) in relation to the dayspost-injury. (**d**) Confocal images of layer II/III of the cortex below the concussion impact point stained for neurotrace (cyan) and dapi (red) and quantification of the number of cells per field of views (FOVs). Scale bar equals 30μm. (**e**) Confocal images of layer II/III of the cortex below the concussion impact point stained for activated caspase-3 (a-casp3; green) and dapi (red) and quantification of the number of cells stained for caspase-3 per field of views (FOVs). Scale bar equals 30μm. n=12 mice for the apnea, righting reflex and weight analysis. n=11 for the control group for the NT435 and casp3 stainings. n=4-5 for all experimental groups for the NT435 and casp3 stainings. All data (presented as mean ±SEM) were tested with the Shapiro-Wilk test and are normally distributed except for the righting reflex. Comparisons were then made for parametric datasets using one-way ANOVA followed by Dunnett’s multiple comparison test and for unparametric datasets with a Kruskall-Wallis test followed by-Dunn’s test. Ctrl: Control; a-casp3: activated capsase 3; c:concussion. Ctrl: Control; wk: week.


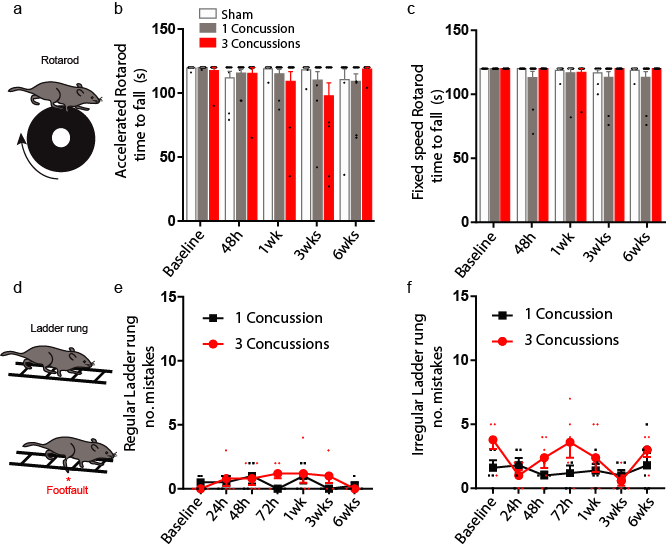


**Supplementary Figure 2. Motor performance does not change following single or repetitive concussion.** (**a**) Schematic representation of the rotarod test. (**b**) Quantification of the time to fall in the accelerated rotarod in sham (white box), single concussion (grey box) and repetitive concussions (red box) at different time points following injury (48hrs, 1wk, 3wks, 6wks). (**c**) Quantification of the time to fall in the fixed speed rotarod in sham (white box), single concussion (grey box) and repetitive concussions (red box) at different time points following injury (48hrs, 1wk, 3wks, 6wks). (**d**) Schematic representation of the ladder rung test. (**e**) Quantification of the number of mistakes after single concussion (black line) and repetitive concussions (red line) at different time points following injury (48hrs, 1wk, 3wks, 6wks) for the regular ladder rung. (**f**) Quantification of the number of mistakes after single concussion (black line) and repetitive concussions (red line) at different time points following injury (48hrs, 1wk, 3wks, 6wks) for the irregular ladder rung. Data sets were normally distributed (Shapiro-Wilk test) and were analyzed with a two-way ANOVA and post-hoc test.


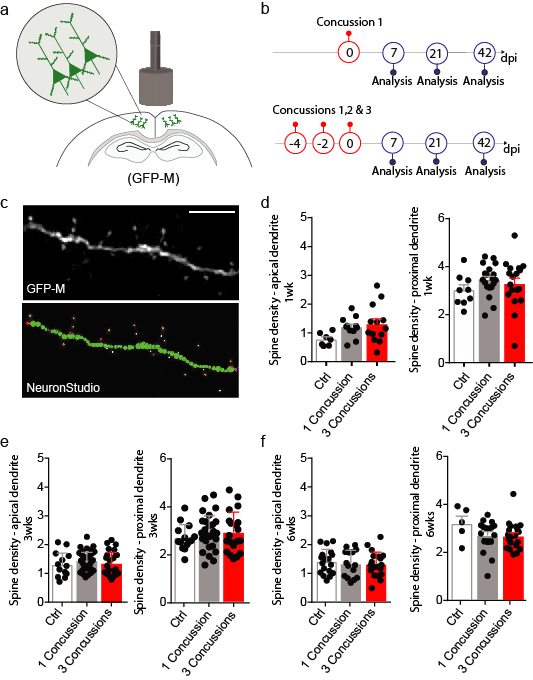


**Supplementary Figure 3. Single and repetitive concussions do not trigger any changes in spine density.** (**a**) Scheme of the experimental design (green: GFP positive neurons). (**b**) Timeline of the experiment. (**c**) Confocal images of a dendritic stretch (top) and representative example illustrating the spine quantification using Neuronstudio (bottom). Scale bar equals 5 μm. (**d**) Quantification of the spine density in control and following single and repetitive concussions at 1 week post-injury in the apical tuft and in the proximal dendritic stretch. n=20-28 independent dendritic stretches from 4 to 5 mice for apical and n = 20-28 independent dendritic stretches from 4 to 5 mice for proximal. (**e**) Quantification of the spine density in control and following single and repetitive concussions at 3 weeks post-injury in the apical tuft and in the proximal dendritic stretch. n =21-30 independent dendritic stretches from 4 to 5 mice for apical and n = 15-28 independent dendritic stretches from 4 to 5 mice for proximal. (**f**) Quantification of the spine density in control and following single and repetitive concussions at 6 weeks post-injury in the apical tuft and in the proximal dendritic stretch. n = 15-28 independent dendritic stretches from 4 to 5 mice for Apical and n = 17-28 independent dendritic stretches from 4 to 5 mice for Proximal. Data are presented as mean±SEM. Ctrl: Control; wk: week.


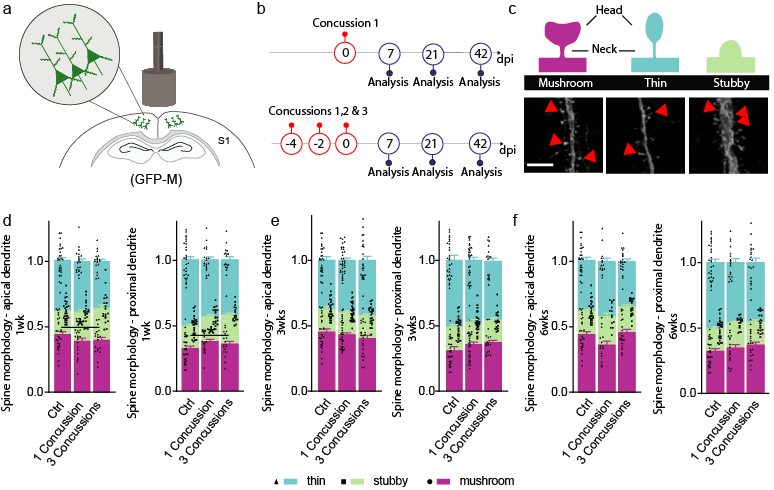


**Supplementary Figure 4. Pyramidal cortical neurons display subtle changes in spine morphology following repetitive concussions.** (**a**) Scheme of the experimental design (green: GFP positive neurons). (**b**) Timeline of the experiment. (**c**) Schematic representation of spine morphology and confocal images of mushroom, thin and stubby spines. Red arrowheads represent the examples of the respective spine type. Scale bar: 5 μm. (**d**) Quantification (mean ± SEM) of the fraction of mushroom, thin and stubby spines on apical tuft and proximal dendrites of layer II/III callosal neurons at 1 week post-injury. n = 20-28 independent dendritic stretches from 4-5 mice for Apical and n = 20-28 independent dendritic stretches from 4-5 mice for Proximal. Apical stubby spines: p = 0.0286 Ctrl vs 3 Concussion, One-way ANOVA and Dunnett’s test. Proximal stubby spines: p = 0.0317 Ctrl vs 3 concussions, One-way ANOVA and Dunnett’s test. (**e**) Quantification (mean ± SEM) of the fraction of mushroom, thin and stubby spines on apical tuft and proximal dendrites of layer II/III callosal neurons at 3 weeks post-injury. n = 24-33 independent dendritic stretches from 4-5 mice for Apical and n = 21-30 independent dendritic stretches from 4-5 mice for Proximal. (**f**) Quantification (mean ± SEM) of the fraction of mushroom, thin and stubby spines on apical tuft and proximal dendrites of layer II/III callosal neurons at 6 weeks post-injury. n = 15-28 independent dendritic stretches from 4 - 5 mice for Apical and n = 17-28 independent dendritic stretches from 4 to 5 mice for Proximal. Ctrl: Control; wk: week.
