# Supplementary material for: Repetitive concussions promote microglia-mediated engulfment of presynaptic excitatory input associated with cognitive dysfunction
Source: Commun Biol. 2025 Feb 28;8:335. doi: 10.1038/s42003-025-07729-1 (PMC11871131; doi:10.1038/s42003-025-07729-1)
Supplement: Supplementary file 2 — Reporting summary [file 42003_2025_7729_MOESM2_ESM.pdf]

nature portfolio

Corresponding author(s): Bareyre FM

Last updated by author(s): 30.12.2024

Reporting Summary

Nature Portfolio wishes to improve the reproducibility of the work that we publish. This form provides structure for consistency and transparency in reporting. For further information on Nature Portfolio policies, see our Editorial Policies and the Editorial Policy Checklist.

Statistics

For all statistical analyses, confirm that the following items are present in the figure legend, table legend, main text, or Methods section.

n/a	Confirmed	
☐	☒	The exact sample size (*n*) for each experimental group/condition, given as a discrete number and unit of measurement
☐	☒	A statement on whether measurements were taken from distinct samples or whether the same sample was measured repeatedly
☐	☒	The statistical test(s) used AND whether they are one- or two-sided<br>*Only common tests should be described solely by name; describe more complex techniques in the Methods section.*
☒	☐	A description of all covariates tested
☒	☐	A description of any assumptions or corrections, such as tests of normality and adjustment for multiple comparisons
☐	☒	A full description of the statistical parameters including central tendency (e.g. means) or other basic estimates (e.g. regression coefficient) AND variation (e.g. standard deviation) or associated estimates of uncertainty (e.g. confidence intervals)
☐	☒	For null hypothesis testing, the test statistic (e.g. *F*, *t*, *r*) with confidence intervals, effect sizes, degrees of freedom and *P* value noted<br>*Give P values as exact values whenever suitable.*
☒	☐	For Bayesian analysis, information on the choice of priors and Markov chain Monte Carlo settings
☒	☐	For hierarchical and complex designs, identification of the appropriate level for tests and full reporting of outcomes
☒	☐	Estimates of effect sizes (e.g. Cohen's *d*, Pearson's *r*), indicating how they were calculated

Our web collection on statistics for biologists contains articles on many of the points above.

Software and code

Policy information about availability of computer code

Data collection	Data were collected using one GoPro 8 camera at 120 frames per second for behavioral data. Data were collected using a confocal microscope FV1000 Olympus (60x oil objective NA 1.35, zoom of 3 and resolution of 1024×1024) and a confocal microscope Leica SP8X WLL.
Data analysis	Data were analyzed using Behavioral Observation Research Interactive Software (BORIS), Imaris software 9.6.0 version (BitPlane), Neuronstudio (version 0.9.92), MATLAB (MathWorks) and Fiji (ImageJ, v.1.52 h).

For manuscripts utilizing custom algorithms or software that are central to the research but not yet described in published literature, software must be made available to editors and reviewers. We strongly encourage code deposition in a community repository (e.g. GitHub). See the Nature Portfolio guidelines for submitting code & software for further information.

Data

Policy information about availability of data

All manuscripts must include a data availability statement. This statement should provide the following information, where applicable:

- Accession codes, unique identifiers, or web links for publicly available datasets
- A description of any restrictions on data availability
- For clinical datasets or third party data, please ensure that the statement adheres to our policy

All data are available in the manuscript or in the supplementary materials. Raw data is availbale upon reasonable request to the corresponding author. All codes are provided.

Human research participants

Policy information about studies involving human research participants and Sex and Gender in Research.

Reporting on sex and gender	*Use the terms sex (biological attribute) and gender (shaped by social and cultural circumstances) carefully in order to avoid confusing both terms. Indicate if findings apply to only one sex or gender; describe whether sex and gender were considered in study design whether sex and/or gender was determined based on self-reporting or assigned and methods used. Provide in the source data disaggregated sex and gender data where this information has been collected, and consent has been obtained for sharing of individual-level data; provide overall numbers in this Reporting Summary. Please state if this information has not been collected. Report sex- and gender-based analyses where performed, justify reasons for lack of sex- and gender-based analysis.*
Population characteristics	*Describe the covariate-relevant population characteristics of the human research participants (e.g. age, genotypic information, past and current diagnosis and treatment categories). If you filled out the behavioural & social sciences study design questions and have nothing to add here, write "See above."*
Recruitment	*Describe how participants were recruited. Outline any potential self-selection bias or other biases that may be present and how these are likely to impact results.*
Ethics oversight	*Identify the organization(s) that approved the study protocol.*

Note that full information on the approval of the study protocol must also be provided in the manuscript.

Field-specific reporting

Please select the one below that is the best fit for your research. If you are not sure, read the appropriate sections before making your selection.

☒ Life sciences ☐ Behavioural & social sciences ☐ Ecological, evolutionary & environmental sciences

For a reference copy of the document with all sections, see nature.com/documents/nr-reporting-summary-flat.pdf

Life sciences study design

All studies must disclose on these points even when the disclosure is negative.

Sample size	No statistical methods were used to pre-determine sample size but our sample sizes are similar to those reported in previous publications. Bradley, P. M. et al. J. Exp. Med. 216, 2503–2514 (2019). Van Steenbergen et al., J. Exp. Med 220 (3): e20220615 (2023) and Empl et al., Nat. Comm. 13:2659 (2022).
Data exclusions	Pre-established exclusion criteria were used in this study. For TBI, mice were included only if they showed a righting reflex and apnea compatible with mild injuries.
Replication	All experiments in this study include at least 5 biological replicates for anatomical data and at least 8 replicates for behavioral data. The number of replicates (n) is mentioned in the text or figure legend.
Randomization	Mice were injured and followed over time for single or repetitive concussions. No randomization was necessary as there is no control or treatment groups. The mice were tested pre-injury and then injured and tested again at different time points post-injury (behavior). Otherwise there was no randomization necessary.
Blinding	Data collection and analysis was performed blind to the conditions of the experiment, unless this was not possible (for exemple, behavioral recording with the GoPro camera pre/post-injury).

Reporting for specific materials, systems and methods

We require information from authors about some types of materials, experimental systems and methods used in many studies. Here, indicate whether each material, system or method listed is relevant to your study. If you are not sure if a list item applies to your research, read the appropriate section before selecting a response.

Materials & experimental systems

n/a	Involved in the study
☐	☒ Antibodies
☒	☐ Eukaryotic cell lines
☒	☐ Palaeontology and archaeology
☐	☒ Animals and other organisms
☒	☐ Clinical data
☒	☐ Dual use research of concern

Methods

n/a	Involved in the study
☒	☐ ChIP-seq
☒	☐ Flow cytometry
☒	☐ MRI-based neuroimaging

Antibodies

Antibodies used	anti-rabbit anti-ionized calcium-binding adaptor molecule-1 [IBA-1] (1/500, Wako, N°019-19741). Alexa-594 donkey anti-rabbit (1/500,ThermoFisher, A21207). guinea pig anti-IBA1 (1/500, Synaptic Systems, N°234 004), rabbit anti-VGLUT1/2 (1/500, Synaptic Systems, N°135 503), rat anti-CD68 (1/500, Abcam, N°ab53444). rabbit anti-IBA1 (1/500, Wako, N°019-19741), guinea pig anti-VGAT (1/500, Synaptic Systems, N°131 308), rat anti-CD68 (1/500, Abcam, N°ab53444). anti-guinea pig Alexa Fluor (AF) 633 (1/500, Sigma Aldrich, N°SAB4600129), anti-rat AF594 (1/500, ThermoFisher, N° A21209), anti-rabbit AF488 (1/500, ThermoFisher, N°A21206), anti-rabbit AF647 (1/500, ThermoFisher, N°A32795), anti-guinea pig Cy3 (1/500, Jackson Immunoresearch, N°706-165-148), anti-rat AF488 (1/500, Abcam, N°ab150153). Caspase 3 antibody (1/250,Abcam, ab2302) coupled with an anti-rabbit AF647 (1/500, ThermoFisher, A32795).
Validation	All published and commercially availble antibodies.

Animals and other research organisms

Policy information about studies involving animals; ARRIVE guidelines recommended for reporting animal research, and Sex and Gender in Research

Laboratory animals	C57Bl6J background and GFP-M mice, eight to twelve weeks-old, were used for this study.
Wild animals	No wild animals were used in this paper.
Reporting on sex	mixed gender (predominantly female: 80%).
Field-collected samples	No field-collected samples were used here.
Ethics oversight	All animal experiments were performed in accordance with the regulations of the local animal committee of the state of Bavaria (Regierung von Oberbayern).

Note that full information on the approval of the study protocol must also be provided in the manuscript.
